# Supplementary material for: Molecular Biointerface Characterization for an Implanted Medical Device Using Cryogenic Orbitrap Secondary Ion Mass Spectrometry (Cryo-OrbiSIMS)
Source: ACS Appl Mater Interfaces. 2026 Feb 5;18(6):9543–58. doi: 10.1021/acsami.5c24925 (PMC12926936; doi:10.1021/acsami.5c24925)
Supplement: Supplementary file 1 [file am5c24925_si_001.pdf]

Supporting Information

Molecular Biointerface Characterisation for an Implanted Medical Device Using Cryogenic Orbitrap Secondary Ion Mass Spectrometry (Cryo-OrbiSIMS)

Akmal Hidayat Bin Sabri [a], Kei F. Carver Wong[a], Anna M. Kotowska[a], Leanne E Fisher[a], Jeni Luckett[b], Jimiama Mafeni Mase[c], Lisa Kämmerling[b], Grazziela Figueredo[c,d], David J. Scurr[a], Amir M. Ghaemmaghami[b], Morgan R. Alexander [a]*.

[a]School of Pharmacy, Faculty of Science, University of Nottingham, University Park, Nottingham, NG7 2RD, UK

[b]School of Life Sciences, Faculty of Medicine and Health Science, University of Nottingham, University Park, Nottingham, NG7 2RD, UK

[c]School of Computer Science, University of Nottingham, University Park, Nottingham, NG7 2RD, UK

[d]Health Data Science, Faculty of Medicine & Health Sciences, University of Nottingham, University Park, Nottingham, NG7 2RD, UK

*Corresponding author

Corresponding author email: morgan.alexander@nottingham.ac.uk

Data Analysis and Machine Learning

1. Data Pre-processing and Normalisation

The original dataset includes 60 PDMS samples (n=30 per time point) containing 282 attributes, considering only instances obtained under cryogenic temperature. OrbiSIMS spectra and profile that are collected after 24h and 28 days were included. These timeframes were defined as the dependent variables for the modelling exercise. The entire analytics was conducted using Helix[52], an open-source tool for data analysis and Machine Learning modelling and interpretation developed by our team. As the dataset is high dimensional and sparse, Least Absolute Shrinkage and Selection Operator (LASSO) was employed to eliminate less important variables before modelling. We also removed those variables with correlation above 0.95. This resulted in 54 independent variables to be used for modelling. Shapiro-Wilk and Kolgomorov-Smirnov tests reveal that most variables do not follow a normal distribution (**Table S3**).

2. Modelling

As the dataset is not normally distributed, we modelled the data using logistic regression and compared the results with non-linear Machine Learning approaches, namely, Random Forest, Support Vector Machine and XGBoost, using 5-fold cross-validation and 20% of the data for test. Hyperparameter search was conducted using grid search and results were collected for the models with best parameters. Improved results were obtained with the non-normalised data. Results are shown in **Table S4**. Logistic Regression and Random Forest produced the best classification results.

3. Post-Training Interpretation

To understand variable importance for logistic regression, we calculated the variable average beta coefficients across the 5 folds used for cross validation and their p-value to assess their significance, as shown in **Table S5**. A p-value smaller than 0.05 suggests statistical significance.

We use Shapley Additive explanation (SHAP) values to the logistic regression to further understand variable contributions to the outcome (**Table S4**). The variable 255.2328 appears to have the highest impact in the model, and its prevalence (high values represented as blue dots) affect more strongly data collected for 28 days. In the figure negative shap values indicate stronger correlation with 24h endpoint and positive correlate with 28 days data.

4. Machine Learning Interpretation

We utilised an ensemble post-training feature interpretation approach, implemented in Helix and detailed in the references provided in the comment, to better understand how variables influence both linear and non-linear model outcomes. Given the overall high accuracy achieved, we computed the mean and majority vote of the most important variables identified across all models (logistic regression, random forest, support vector machines, and XGBoost).

Overall, there was consensus regarding the most important variables, although differences in ranking emerged depending on the ensemble method employed. The **mean ensemble** quantifies variable importance across all machine learning models using two post-training methods: SHAP and Permutation Importance. In contrast, **majority voting** ranks variables based on their importance within each model and feature importance calculation, with the final ranking determined by the most frequently selected variables across methods.

Table S1. Tabulated output from the SIMS-MFP software showing the putative lipid assignments along with observed mass (m/z), mass deviation, chemical formula, molecular assignment and adduct. All assignments were compared against the Lipid Maps Structural Database®.

mass	error	Formula	Assignment	Adduct
549.4879	0.298	$C_{35}H_{65}O_4^+$	DG O-32:3/MG 32:3/MG O-32:4;O	[M+H]
573.487	-1.284	$C_{37}H_{65}O_4^+$	CE 10:0;O2/DG O-34:5/MG 34:5/MG O-34:6;O	[M+H]
597.4869	-1.399	$C_{39}H_{65}O_4^+$	CE 12:2;O2/DG O-36:7	[M+H]
551.5039	0.932	$C_{35}H_{67}O_4^+$	DG O-32:2/MG 32:2/MG O-32:3;O	[M+H]
575.5039	0.893	$C_{37}H_{67}O_4^+$	DG O-34:4/MG 34:4/MG O-34:5;O	[M+H]
599.5036	0.357	$C_{39}H_{67}O_4^+$	CE 12:1;O2/DG O-36:6	[M+H]
666.4845	-1.802	$C_{38}H_{69}NO_6P^+$	CerP 38:5;O2/LPC O-30:6/LPE O-33:6	[M+H]
601.5191	0.106	$C_{39}H_{69}O_4^+$	CE 12:0;O2/DG O-36:5	[M+H]
621.4858	0.697	$C_{34}H_{70}O_7P^+$	LPA 31:0/LPA O-31:1;O/PA O-31:0	[M+H]
603.5353	1.017	$C_{39}H_{71}O_4^+$	DG O-36:4	[M+H]
623.5013	0.454	$C_{34}H_{72}O_7P^+$	LPA O-31:0;O	[M+H]
725.5576	1.914	$C_{42}H_{77}O_9^+$	TG 39:2;O3	[M+H]
756.5527	-1.429	$C_{42}H_{79}NO_8P^+$	CerP 42:4;O4/LPC 34:4;O/PC 34:3/PC O-34:4;O/ PE 37:3/PE O-37:4;O	[M+H]
780.5524	-1.769	$C_{44}H_{79}NO_8P^+$	CerP 44:6;O4/PC 36:5/PC O-36:6;O/PE 39:5/ PE O-39:6;O	[M+H]
542.4906	-0.275	$C_{34}H_{65}NO_2Na^+$	Cer 34:2;O/NAE 32:2	[M+Na]
621.4858	0.756	$C_{39}H_{66}O_4Na^+$	CE 12:1;O2/DG O-36:6	[M+Na]
623.5013	0.513	$C_{39}H_{68}O_4Na^+$	CE 12:0;O2/DG O-36:5	[M+Na]
577.517	0.641	$C_{35}H_{70}O_4Na^+$	DG O-32:0/MG 32:0/MG O-32:1;O	[M+Na]
666.4845	1.808	$C_{36}H_{70}NO_6PNa^+$	CerP 36:2;O2/LPC O-28:3/LPE O-31:3	[M+Na]
625.5176	1.551	$C_{39}H_{70}O_4Na^+$	DG O-36:4	[M+Na]
725.5576	1.111	$C_{39}H_{79}N_2O_6PNa^+$	PE-Cer 37:1;O2/SM 34:1;O2	[M+Na]
773.5296	-0.913	$C_{40}H_{79}O_{10}PNa^+$	BMP 34:0/LPG 34:1;O/PG 34:0/PG O-34:1;O	[M+Na]
756.5527	1.751	$C_{40}H_{80}NO_8PNa^+$	CerP 40:1;O4/LPC 32:1;O/LPS O-34:1/PC 32:0/ PC O-32:1;O/PE 35:0/PE O-35:1;O	[M+Na]
780.5524	1.313	$C_{42}H_{80}NO_8PNa^+$	CerP 42:3;O4/LPC 34:3;O/PC 34:2/PC O-34:3;O/ PE 37:2/PE O-37:3;O	[M+Na]
690.4369	-0.937	$C_{34}H_{69}NO_8SK^+$	NAT 32:0;O4	[M+K]
666.4845	-1.975	$C_{40}H_{69}NO_4K^+$	CAR 33:5/Cer 40:6;O3	[M+K]
621.4858	0.512	$C_{36}H_{70}O_5K^+$	DG 33:0/DG O-33:1;O/MG 33:1;O/TG O-33:0	[M+K]
623.5013	0.269	$C_{36}H_{72}O_5K^+$	DG O-33:0;O/MG 33:0;O	[M+K]
756.5527	-1.581	$C_{44}H_{79}NO_6K^+$	ACer 44:4;O4/Cer 44:5;O5	[M+K]
780.5524	-1.917	$C_{46}H_{79}NO_6K^+$	ACer 46:6;O4	[M+K]

mass	error	Formula	Assignment	Adduct
227.2016	-0.237	$C_{14}H_{27}O_2^-$	FA 14:0	[M-H]
299.2014	-0.849	$C_{20}H_{27}O_2^-$	ST 20:3;O2	[M-H]
253.2174	0.380	$C_{16}H_{29}O_2^-$	FA 16:1	[M-H]
301.2172	-0.345	$C_{20}H_{29}O_2^-$	FA 20:5/ST 20:2;O2	[M-H]
255.2328	-0.603	$C_{16}H_{31}O_2^-$	FA 16:0	[M-H]
279.2326	-1.267	$C_{18}H_{31}O_2^-$	FA 18:2	[M-H]
303.2327	-0.837	$C_{20}H_{31}O_2^-$	FA 20:4/ST 20:1;O2	[M-H]
327.2326	-1.081	$C_{22}H_{31}O_2^-$	FA 22:6/ST 22:3;O2	[M-H]
269.2488	0.728	$C_{17}H_{33}O_2^-$	FA 17:0	[M-H]
281.2486	-0.014	$C_{18}H_{33}O_2^-$	FA 18:1	[M-H]
305.2483	-0.996	$C_{20}H_{33}O_2^-$	FA 20:3/ST 20:0;O2	[M-H]
329.2484	-0.619	$C_{22}H_{33}O_2^-$	FA 22:5/ST 22:2;O2	[M-H]
283.2642	-0.190	$C_{18}H_{35}O_2^-$	FA 18:0	[M-H]
331.2639	-1.068	$C_{22}H_{35}O_2^-$	FA 22:4/ST 22:1;O2	[M-H]
391.2251	-1.022	$C_{19}H_{36}O_6P^-$	LPA O-16:2	[M-H]
420.2601	-0.459	$C_{20}H_{38}NO_8^-$	CAR 13:0;O4	[M-H]
417.2411	-0.120	$C_{21}H_{38}O_6P^-$	LPA O-18:3	[M-H]
359.2954	-0.428	$C_{24}H_{39}O_2^-$	FA 24:4/ST 24:1;O2	[M-H]
419.2574	1.431	$C_{21}H_{40}O_6P^-$	LPA O-18:2	[M-H]
437.2675	0.309	$C_{21}H_{42}O_7P^-$	LPA 18:0/LPA O-18:1;O	[M-H]
436.2835	0.346	$C_{21}H_{43}NO_6P^-$	LPC O-13:1/LPE O-16:1	[M-H]
462.2992	0.435	$C_{23}H_{45}NO_6P^-$	LPC O-15:2/LPE O-18:2	[M-H]
464.3142	-0.967	$C_{23}H_{47}NO_6P^-$	LPC O-15:1/LPE O-18:1	[M-H]
480.3098	0.492	$C_{23}H_{47}NO_7P^-$	LPC 15:0/LPC O-15:1;O/LPE 18:0/LPE O-18:1;O	[M-H]
588.3738	1.667	$C_{34}H_{54}NO_5S^-$	NAT 32:7;O	[M-H]
616.4721	1.543	$C_{34}H_{67}NO_6P^-$	CerP 34:1;O2/LPC O-26:2/LPE O-29:2	[M-H]
905.3844	-1.658	$C_{42}H_{67}O_{17}P_2^-$	PIP 33:7;O	[M-H]
642.4864	-0.621	$C_{36}H_{69}NO_6P^-$	CerP 36:2;O2/LPC O-28:3/LPE O-31:3	[M-H]
722.5129	-0.158	$C_{41}H_{73}NO_7P^-$	CerP 41:5;O3/LPC 33:5/LPC O-33:6;O/PC O-33:5/PE O-36:5	[M-H]
746.514	1.321	$C_{43}H_{73}NO_7P^-$	PC O-35:7/PE O-38:7	[M-H]
748.5296	1.251	$C_{43}H_{75}NO_7P^-$	CerP 43:6;O3/PC O-35:6/PE O-38:6	[M-H]
723.517	-1.603	$C_{38}H_{76}O_{10}P^-$	LPG 32:0;O/PG O-32:0;O	[M-H]
687.5443	-0.506	$C_{38}H_{76}N_2O_6P^-$	PE-Cer 36:1;O2/SM 33:1;O2	[M-H]

747.517	-1.552	$C_{40}H_{76}O_{10}P^-$	BMP 34:1/LPG 34:2;O/PG 34:1/PG O-34:2;O	[M-H]
858.5235	1.691	$C_{44}H_{76}NO_{15}^-$	Hex2Cer 32:4;O4	[M-H]
718.5382	-1.432	$C_{39}H_{77}NO_8P^-$	CerP 39:1;O4/LPC 31:1;O/LPE 34:1;O/LPS O-33:1/PC 31:0/PC O-31:1;O/PE 34:0/PE O-34:1;O	[M-H]
742.539	-0.308	$C_{41}H_{77}NO_8P^-$	CerP 41:3;O4/LPC 33:3;O/PC 33:2/PC O-33:3;O/PE 36:2/PE O-36:3;O	[M-H]
750.5438	-0.685	$C_{43}H_{77}NO_7P^-$	CerP 43:5;O3/PC O-35:5/PE O-38:5	[M-H]
766.5398	0.745	$C_{43}H_{77}NO_8P^-$	CerP 43:5;O4/PC 35:4/PC O-35:5;O/PE 38:4/PE O-38:5;O	[M-H]
994.4794	1.198	$C_{46}H_{77}NO_{20}P^-$	MIPC 34:6;O6	[M-H]
857.5185	-0.064	$C_{45}H_{78}O_{13}P^-$	PI 36:4/PI O-36:5;O	[M-H]
744.555	0.163	$C_{41}H_{79}NO_8P^-$	CerP 41:2;O4/LPC 33:2;O/PC 33:1/PC O-33:2;O/PE 36:1/PE O-36:2;O	[M-H]
886.5531	-0.280	$C_{46}H_{80}NO_{15}^-$	Hex2Cer 34:4;O4	[M-H]
883.5344	0.221	$C_{47}H_{80}O_{13}P^-$	PI 38:5/PI O-38:6;O	[M-H]
778.5754	-0.275	$C_{45}H_{81}NO_7P^-$	CerP 45:5;O3/PC O-37:5/PE O-40:5	[M-H]
794.5717	1.474	$C_{45}H_{81}NO_8P^-$	CerP 45:5;O4/PC 37:4/PC O-37:5;O/PE 40:4/PE O-40:5;O	[M-H]
861.5491	-0.876	$C_{45}H_{82}O_{13}P^-$	PI 36:2/PI O-36:3;O	[M-H]
885.549	-0.965	$C_{47}H_{82}O_{13}P^-$	PI 38:4/PI O-38:5;O	[M-H]
814.5983	1.910	$C_{45}H_{85}NO_9P^-$	CerP 45:3;O5/PC 37:2;O/PE 40:2;O/PS O-39:2	[M-H]
910.5677	1.610	$C_{45}H_{85}NO_{15}P^-$	PI-Cer 39:2;O6	[M-H]
833.5926	1.530	$C_{45}H_{86}O_{11}P^-$	PG 39:1;O	[M-H]
813.6008	-0.854	$C_{46}H_{86}O_9P^-$	PA 43:2;O/PG O-40:3	[M-H]
711.6513	0.683	$C_{44}H_{87}O_6^-$	DG O-41:0;O2	[M-H]
795.6392	0.820	$C_{46}H_{88}N_2O_6P^-$	PE-Cer 44:3;O2/SM 41:3;O2	[M-H]
810.6464	-0.071	$C_{47}H_{88}NO_9^-$	HexCer 41:2;O3	[M-H]
790.6345	1.734	$C_{44}H_{89}NO_8P^-$	CerP 44:0;O4/PC O-36:0;O/PE O-39:0;O	[M-H]
797.6543	0.128	$C_{46}H_{90}N_2O_6P^-$	PE-Cer 44:2;O2/SM 41:2;O2	[M-H]
952.5239	1.122	$C_{47}H_{83}NO_{14}SCl^-$	SHexCer 41:5;O5	[M+Cl]
1013.492	-1.049	$C_{48}H_{84}O_{16}P_2Cl^-$	PIP 39:5	[M+Cl]
790.6345	1.512	$C_{45}H_{89}NO_7Cl^-$	ACer 45:0;O5/Cer 45:1;O6	[M+Cl]

Table S2. Tabulated output from the SIMS-MFP software showing the putative biomolecular assignments along with observed mass (m/z), mass deviation, chemical formula, molecular assignment and adduct. All assignments were compared against the Human Metabolome Database®.

mass	error	formula	Assignment	Adduct

206.9961	1.512	$C_6H_7O_6S$	Pyrogallol-2-O-sulphate	[M+H]
130.0653	1.345	C_9H_8N	leucoline	[M+H]
173.0269	1.202	$C_7H_9O_3S$	p-Toluenesulfonic acid	[M+H]
168.0588	-1.155	$C_7H_{10}N_3S$	6-Methyl-7,8-dihydroimidazo[1,5-c]pyrimidine-5(6H)-thione	[M+H]
91.0574	-2.165	$C_4H_{11}S$	butanethiol	[M+H]
107.0523	-1.981	$C_4H_{11}OS$	methionol	[M+H]
179.0553	1.586	$C_6H_{11}O_6$	L-Gulono-14-lactone/ D-Glucono-14-lactone	[M+H]
86.0966	2.031	$C_5H_{12}N$	Piperidine	[M+H]
109.1013	1.136	C_8H_{13}	(Z)-1,3-Octadiene	[M+H]
265.0378	0.558	$C_9H_{13}O_7S$	3-Methoxy-4-Hydroxyphenylglycolsulfate	[M+H]
184.0736	1.515	$C_5H_{15}NO_4P$	Choline phosphate	[M+H]
250.0085	-0.840	$C_5H_{10}NO_7PNa$	Î³-Glutamyl phosphate	[M+Na]
270.0142	1.500	$C_8H_{10}NO_6PNa$	Pyridoxal phosphate	[M+Na]
184.0736	1.716	$C_{10}H_{11}NONa$	Tryptophol	[M+Na]
759.5121	-1.189	$C_{41}H_{72}N_2O_9Na$	oceanalin A	[M+Na]
773.5296	-0.913	$C_{40}H_{79}O_0PNa$	1-hexadecanoyl-2-octadecanoyl-sn-glycero-3-phospho-(1 -sn-glycerol)	[M+Na]
124.9997	-1.897	$C_4H_6O_2K$	3-Butenoic acid/ Biacetyl/ butyrolactone/ oxolan-3-one	[M+K]
157.016	-1.625	$C_7H_6N_2K$	pyrrolo[1,2-a]pyrazine	[M+K]
216.99	1.073	$C_9H_6O_4K$	6,7-Dihydroxycoumarin	[M+K]
231.0055	0.359	$C_{10}H_8O_4K$	Scopoletin	[M+K]
211.0271	1.326	$C_{10}H_8N_2OK$	4-Hydroxy-1H-indole-3-acetonitrile	[M+K]
192.9756	1.145	$C_4H_{10}O_2S_2K$	2-hydroxyethyldisulfide	[M+K]
237.9879	0.705	$C_4H_{10}NO_6PK$	(S)-2-Amino-3-hydroxybutanoic acid 3-phosphate	[M+K]
265.9829	0.951	$C_5H_{10}NO_7PK$	Î³-Glutamyl phosphate	[M+K]
249.016	0.072	$C_{10}H_{10}O_5K$	3-(4-Hydroxy-3-methoxyphenyl)-2-oxiranecarboxylic acid	[M+K]
228.0385	1.692	$C_6H_{11}N_3O_4K$	gly-asn	[M+K]
227.0427	-0.727	$C_7H_{12}N_2O_4K$	N-Acetylglutamine	[M+K]
184.0736	0.890	$C_7H_{15}NO_2K$	4-Trimethylammoniobutanoate/4-Trimethylammoniobutanoate/ g-Butyrobetaine	[M+K]

mass	error	formula	Assignment	Adduct
82.9958	-3.553	$C_4H_3S^-$	Thiophene	[M-H]
83.0136	-3.059	$C_4H_3O_2^-$	2[5H]-furanone	[M-H]

99.0086	-1.706	$C_4H_3O_3^-$	Succinic anhydride	[M-H]
111.0199	-0.918	$C_4H_3N_2O_2^-$	Uracil/ 4-CARBOXYPYRAZOLE	[M-H]
111.0087	-0.621	$C_5H_3O_3^-$	2-Furoic acid	[M-H]
127.0039	1.702	$C_5H_3O_4^-$	5-Hydroxy-2-furoic acid	[M-H]
123.0198	-1.641	$C_5H_3N_2O_2^-$	Pyrazinoate/ Pyrazinoic acid	[M-H]
135.031	-1.740	$C_5H_3N_4O^-$	Hypoxanthine	[M-H]
167.0208	-1.586	$C_5H_3N_4O_3^-$	Urate/ Uric Acid	[M-H]
110.036	0.128	$C_4H_4N_3O^-$	Cytosine	[M-H]
126.0308	-0.801	$C_4H_4N_3O_2^-$	(3r,4s)-Tofacitinib	[M-H]
94.0294	-4.657	$C_5H_4NO^-$	2-pyridone	[M-H]
126.0018	-0.864	$C_5H_4NOS^-$	2-Acetylthiazole	[M-H]
110.0246	-1.390	$C_5H_4NO_2^-$	Pyrrole 2-carboxylate	[M-H]
126.0196	-0.539	$C_5H_4NO_3^-$	4-Hydroxy-1H-pyrrole-2-carboxylic acid	[M-H]
134.047	-1.633	$C_5H_4N_5^-$	Adenine	[M-H]
150.042	-0.892	$C_5H_4N_5O_1^-$	Guanine	[M-H]
122.0246	-1.253	$C_6H_4NO_2^-$	Picolinic acid/ Isonicotinic acid	[M-H]
138.0195	-1.216	$C_6H_4NO_3^-$	4-Nitrophenol	[M-H]
146.0245	-1.732	$C_8H_4NO_2^-$	Indole-5,6-quinone/ Isatin	[M-H]
85.0292	-3.574	$C_4H_5O_2^-$	3-Butenoic acid/ Biacetyl/ butyrolactone/ oxolan-3-one	[M-H]
133.0145	1.888	$C_4H_5O_5^-$	Malate/ (±)-Malic Acid	[M-H]
149.0093	0.913	$C_4H_5O_6^-$	(.+/-.)-Tartaric acid	[M-H]
113.0356	-0.459	$C_4H_5N_2O_2^-$	Dihydrouracil/ Dioxy-creatinine	[M-H]
81.0342	-4.799	$C_5H_5O^-$	2-Methylfuran	[M-H]
113.0244	-0.167	$C_5H_5O_3^-$	3-Oxo-4-pentenoic acid/ 4-Hydroxy-5-methylfuran-3(2H)-one	[M-H]
129.0192	-1.038	$C_5H_5O_4^-$	Itaconate/ Mesaconate/ (Z)-glutaconic acid/ 4,5-dioxovaleric acid	[M-H]
93.0456	-2.385	$C_5H_5N_2^-$	Methylpyrazine	[M-H]
125.0356	-0.415	$C_5H_5N_2O_2^-$	Thymine/ Imidazole-4-acetate	[M-H]
125.0243	-0.951	$C_6H_5O_3^-$	(3E)-2,5-Dioxo-3-hexenal/ Pyrogallol	[M-H]
172.9913	-0.606	$C_6H_5O_4S^-$	Phenol sulfate	[M-H]
173.0094	1.365	$C_6H_5O_6^-$	trans-Aconitate/ L-Dehydroascorbate/ (E)-Aconitic Acid	[M-H]
204.9813	0.318	$C_6H_5O_6S^-$	Pyrogallol-2-O-sulphate	[M-H]
121.0406	-1.131	$C_6H_5N_2O^-$	Nicotinamide	[M-H]
137.0355	-1.108	$C_6H_5N_2O_2^-$	Urocanic acid	[M-H]

121.0294	-0.858	$C_7H_5O_2^-$	Benzoate/ 4-Hydroxybenzaldehyde/ Benzoic acid	[M-H]
117.0457	-1.041	$C_7H_5N_2^-$	pyrrolo[1,2-a]pyrazine	[M-H]
117.0345	-0.760	$C_8H_5O^-$	2,4,6-Octatriyn-1-ol/ Coumarone	[M-H]
145.0293	-1.406	$C_9H_5O_2^-$	Coumarin	[M-H]
84.0452	-3.426	$C_4H_6NO^-$	2-Pyrrolidone	[M-H]
116.0353	-0.154	$C_4H_6NO_3^-$	Aceturic acid	[M-H]
112.0515	-1.213	$C_4H_6N_3O^-$	Creatinine	[M-H]
80.0502	-4.658	$C_5H_6N^-$	methyl pyrrole	[M-H]
112.0405	0.867	$C_5H_6NO_2^-$	1-pyrroline-5-carboxylic acid	[M-H]
128.0352	-0.921	$C_5H_6NO_3^-$	5-Oxoproline/ Pyroglutamic acid	[M-H]
108.0567	-0.193	$C_5H_6N_3^-$	2-Amino-4-methylpyrimidine	[M-H]
124.0515	-1.096	$C_5H_6N_3O^-$	5-Methylcytosine	[M-H]
92.0503	-2.965	$C_6H_6N^-$	Aniline	[M-H]
108.0454	-0.814	$C_6H_6NO^-$	4-Aminophenol	[M-H]
140.0352	-0.842	$C_6H_6NO_3^-$	2-Aminomuconic acid semialdehyde	[M-H]
136.0514	-1.734	$C_6H_6N_3O^-$	Isoniazid	[M-H]
136.0402	-1.491	$C_7H_6NO_2^-$	4-Aminobenzoate/ 4-Aminobenzoic acid	[M-H]
152.0352	-0.775	$C_7H_6NO_3^-$	Mesalazine	[M-H]
116.0505	-0.628	$C_8H_6N^-$	Indole	[M-H]
164.0352	-0.719	$C_8H_6NO_3^-$	3-Hydroxy-2H-indole-5,6(3H,7H)-dione/ Coixol	[M-H]
160.0403	-0.643	$C_9H_6NO_2^-$	3-Carboxyindole	[M-H]
119.0171	-1.049	$C_4H_7O_2S^-$	4-Thiapentanoic acid	[M-H]
99.0563	-0.877	$C_4H_7N_2O^-$	NPYR	[M-H]
115.0512	-0.886	$C_4H_7N_2O_2^-$	1-Nitroso-3-pyrrolidinol/ Succinamide	[M-H]
83.0499	-4.081	$C_5H_7O^-$	Cyclopentanone	[M-H]
115.0401	0.270	$C_5H_7O_3^-$	3-Methyl-2-oxobutanoic acid/ Levulinic acid	[M-H]
127.0512	-0.802	$C_5H_7N_2O_2^-$	5-Nitrilonorvaline	[M-H]
111.045	-1.386	$C_6H_7O_2^-$	Sorbic acid	[M-H]
159.0301	1.265	$C_6H_7O_5^-$	2-Oxoadipate	[M-H]
107.0614	-0.672	$C_6H_7N_2^-$	Phenylhydrazine/ 2,5-Dimethylpyrazine	[M-H]
123.0563	-0.706	$C_6H_7N_2O^-$	2-Methoxy-3-methylpyrazine	[M-H]
139.0512	-0.733	$C_6H_7N_2O_2^-$	1,3-dimethyluracil	[M-H]
155.046	-1.399	$C_6H_7N_2O_3^-$	pentoxyl	[M-H]
91.0551	-2.459	$C_7H_7^-$	Toluene	[M-H]
107.0501	-1.298	$C_7H_7O^-$	Anisole	[M-H]

123.045	-1.251	$C_7H_7O_2^-$	Guaiacol	[M-H]
171.0118	-1.988	$C_7H_7O_3S^-$	p-Toluenesulfonic acid	[M-H]
203.0196	-0.635	$C_7H_7O_7^-$	2,6-Anhydro-6-carboxy-3-deoxyhex-2-enonic acid	[M-H]
119.0614	-0.604	$C_7H_7N_2^-$	2-Methyl-5-vinylpyrazine	[M-H]
135.0563	-0.643	$C_7H_7N_2O^-$	Nicotinyl methylamide	[M-H]
167.046	-1.298	$C_7H_7N_2O_3^-$	N-(1H-Pyrrol-2-ylcarbonyl)glycine	[M-H]
119.0501	-1.167	$C_8H_7O^-$	7-Octene-3,5-diyn-1-ol	[M-H]
135.045	-1.140	$C_8H_7O_2^-$	3-(5-methyl-2-furyl)prop-2-enal	[M-H]
183.0119	-1.311	$C_8H_7O_3S^-$	4-styrenesulfonic acid	[M-H]
131.0613	-1.312	$C_8H_7N_2^-$	1-Methylpyrrolo[1,2-a]pyrazine	[M-H]
147.0449	-1.727	$C_9H_7O_2^-$	2-Chromanone/ Cinnamic acid	[M-H]
143.0502	-0.272	$C_{10}H_7O^-$	1-Naphthol	[M-H]
171.0563	-0.508	$C_{10}H_7N_2O^-$	4-Hydroxy-1H-indole-3-acetonitrile	[M-H]
102.056	-0.518	$C_4H_8NO_2^-$	4-Aminobutanoate/ gamma-Aminobutyric acid	[M-H]
130.0621	-0.776	$C_4H_8N_3O_2^-$	Creatine	[M-H]
114.056	-0.464	$C_5H_8NO_2^-$	L-Proline	[M-H]
130.0508	-1.291	$C_5H_8NO_3^-$	5-Aminolevulinate/ Ac-Ala-OH/ Aminolevulinic acid/ L-Hydroxyproline	[M-H]
110.0723	-0.644	$C_5H_8N_3^-$	1H-Imidazole-4-ethanamine/2-Aminohistamine/ Histamine	[M-H]
110.0611	-0.344	$C_6H_8NO^-$	2-Acetyl-1-pyrroline	[M-H]
126.0559	-1.213	$C_6H_8NO_2^-$	D-1-Piperideine-2-carboxylic acid	[M-H]
142.0508	-1.182	$C_6H_8NO_3^-$	6-Oxo-pipecolinic acid/ trimethadione	[M-H]
138.0671	-1.346	$C_6H_8N_3O^-$	2-Amino-3-(1H-imidazol-4-yl)propanal	[M-H]
154.062	-1.304	$C_6H_8N_3O_2^-$	L-Histidine	[M-H]
170.057	-0.682	$C_6H_8N_3O_3^-$	Metronidazole	[M-H]
106.0662	-0.216	$C_7H_8N^-$	Benzylamine	[M-H]
138.0559	-1.108	$C_7H_8NO_2^-$	Ethyl 2-pyrrolecarboxylate	[M-H]
150.067	-1.905	$C_7H_8N_3O^-$	4-phenylsemicarbazide	[M-H]
134.061	-1.029	$C_8H_8NO^-$	2-Aminoacetophenone/ Acetanilide	[M-H]
130.0661	-0.945	$C_9H_8N^-$	Skatole	[M-H]
146.061	-0.944	$C_9H_8NO^-$	Indole-3-carbidol	[M-H]
162.0559	-0.944	$C_9H_8NO_2^-$	Î³-Oxo-3-pyridinebutanal	[M-H]
158.061	-0.872	$C_{10}H_8NO^-$	Indole-3-acetaldehyde/ N-hydroxy-1-naphthylamine	[M-H]
186.056	-0.284	$C_{11}H_8NO_2^-$	Indoleacrylic acid	[M-H]

89.0426	-4.996	$C_4H_9S^-$	butanethiol	[M-H]
105.0378	-1.522	$C_4H_9OS^-$	methionol	[M-H]
149.0453	-1.670	$C_5H_9O_5^-$	D-Ribose/ D-Arabinose/ 2-deoxy-D-ribonic acid/ D-(+)-Xylose/ D-Ribofuranose/ D-Xylopyranose	[M-H]
165.0407	1.431	$C_5H_9O_6^-$	L-Xylonic acid	[M-H]
113.072	-0.326	$C_5H_9N_2O^-$	3-Amino-2-piperidinone	[M-H]
145.0617	-1.151	$C_5H_9N_2O_3^-$	L-Glutamine	[M-H]
161.0458	1.559	$C_6H_9O_5^-$	2-Hydroxyadipic acid/ Diethylpyrocarbonate/ Meglutol	[M-H]
177.0402	-1.491	$C_6H_9O_6^-$	L-Gulono-14-lactone/ D-Glucono-14-lactone	[M-H]
121.077	-1.007	$C_7H_9N_2^-$	2,4-Diaminotoluene	[M-H]
137.0719	-0.999	$C_7H_9N_2O^-$	2-Ethyl-3-methoxypyrazine	[M-H]
121.0658	-0.734	$C_8H_9O^-$	Phenylethyl alcohol	[M-H]
133.077	-0.916	$C_8H_9N_2^-$	Cyclohexapyrazine	[M-H]
133.0657	-1.420	$C_9H_9O^-$	3-Phenylpropanal/ Cinnamyl alcohol	[M-H]
161.0719	-0.850	$C_9H_9N_2O^-$	norcotinine	[M-H]
193.0616	-1.382	$C_9H_9N_2O_3^-$	4-Aminohippuricacid	[M-H]
116.0716	-0.887	$C_5H_{10}NO_2^-$	L-Valine/ Betaine	[M-H]
144.0776	-1.741	$C_5H_{10}N_3O_2^-$	g-Guanidinobutyrate	[M-H]
241.0115	-1.576	$C_6H_{10}O_8P^-$	1D-myo-Inositol 1,2-cyclic phosphate (Inositol cyclic phosphate)	[M-H]
140.0829	-0.256	$C_6H_{10}N_3O^-$	histidinol	[M-H]
124.0766	-1.514	$C_7H_{10}NO^-$	2-Ethyl-4,5-dimethylthiazole	[M-H]
140.0716	-0.735	$C_7H_{10}NO_2^-$	Ethosuximide	[M-H]
152.0829	-0.236	$C_7H_{10}N_3O^-$	Acetylhistamine	[M-H]
168.0777	-0.898	$C_7H_{10}N_3O_2^-$	N(pi)-Methyl-L-histidine	[M-H]
184.0726	-0.901	$C_7H_{10}N_3O_3^-$	N-(1-Methyl-4-oxo-4,5-dihydro-1H-imidazol-2-yl)alanine	[M-H]
136.0766	-1.381	$C_8H_{10}NO^-$	Tyramine	[M-H]
152.0715	-1.334	$C_8H_{10}NO_2^-$	dopamine	[M-H]
196.073	1.194	$C_8H_{10}N_3O_3^-$	N-Acetyl-DL-histidine	[M-H]
148.0766	-1.269	$C_9H_{10}NO^-$	5-acetyl-2,3-dihydro-1H-pyrrolizine	[M-H]
164.0715	-1.237	$C_9H_{10}NO_2^-$	L-Phenylalanine	[M-H]
176.0827	-1.340	$C_9H_{10}N_3O^-$	NNN	[M-H]
160.0765	-1.799	$C_{10}H_{10}NO^-$	Tryptophol	[M-H]
172.0766	-1.092	$C_{11}H_{10}NO^-$	pyroquilon	[M-H]
188.0716	-0.547	$C_{11}H_{10}NO_2^-$	phensuximide	[M-H]

168.0431	-0.112	$C_4H_{11}NO_4P^-$	P-DMEA	[M-H]
131.0824	-1.540	$C_5H_{11}N_2O_2^-$	L-Ornithine	[M-H]
179.0558	-1.753	$C_6H_{11}O_6^-$	myo-Inositol/ Fructose/ Glucose/ Galactose/ beta-D-Galactopyranose	[M-H]
135.0813	-1.769	$C_9H_{11}O^-$	Nonatrienal/ Mesitol	[M-H]
179.0825	-0.569	$C_9H_{11}N_2O_2^-$	(4-Ethoxyphenyl)urea	[M-H]
195.0773	-1.112	$C_9H_{11}N_2O_3^-$	5-Nitro-2-propoxyaniline	[M-H]
147.0813	-1.624	$C_{10}H_{11}O^-$	Estragole	[M-H]
175.0875	-1.067	$C_{10}H_{11}N_2O^-$	Serotonin	[M-H]
191.0825	-0.533	$C_{10}H_{11}N_2O_2^-$	cotinine N-oxide/ Hydroxycotinine	[M-H]
207.0773	-1.047	$C_{10}H_{11}N_2O_3^-$	L-Kynurenine	[M-H]
203.0825	-0.502	$C_{11}H_{11}N_2O_2^-$	L-Tryptophan	[M-H]
130.0985	-0.660	$C_5H_{12}N_3O^-$	1-(4-Aminobutyl)urea	[M-H]
259.0224	-0.173	$C_6 H_{12}O_9P^-$	D-Fructose 6-phosphate/ D-glucose 6-phosphate/ D-glucopyranose 6-phosphate	[M-H]
130.0872	-1.175	$C_6 H_{12}NO_2^-$	L-Norleucine/ L-Leucine/ L-Isoleucine/ L-Alloisoleucine	[M-H]
126.0923	-1.094	$C_7H_{12}NO^-$	8-Azabicyclo[3.2.1]octan-3-ol	[M-H]
138.0923	-0.999	$C_8H_{12}NO^-$	Norpseudopelletierine	[M-H]
154.0872	-0.992	$C_8H_{12}NO_2^-$	arecoline	[M-H]
166.0871	-1.523	$C_9H_{12}NO_2^-$	3-Methoxytyramine	[M-H]
194.0934	-0.520	$C_9H_{12}N_3O_2^-$	4-(Nitrosoamino)-1-(3-pyridinyl)-1-butanol	[M-H]
210.0883	-0.552	$C_9H_{12}N_3O_3^-$	Zalcitabine	[M-H]
129.1032	-1.060	$C_6H_{13}N_2O^-$	N-Acetylputrescine/ N-Acetylputrescine	[M-H]
145.0981	-1.047	$C_6H_{13}N_2O_2^-$	L-Lysine	[M-H]
169.0981	-0.898	$C_8H_{13}N_2O_2^-$	R-(+)-Etiracetam	[M-H]
165.1032	-0.829	$C_9H_{13}N_2O^-$	2-Isobutyl-3-methoxypyrazine	[M-H]
193.0981	-0.787	$C_{10}H_{13}N_2O_2^-$	6-hydroxypseudooxynicotine	[M-H]
209.093	-0.798	$C_{10}H_{13}N_2O_3^-$	Aprobarbital	[M-H]
189.103	-1.782	$C_{11}H_{13}N_2O^-$	5-Methoxytryptamine	[M-H]
140.1079	-1.341	$C_8H_{14}NO^-$	Isopelletierine	[M-H]
196.1089	-1.279	$C_9H_{14}N_3O_2^-$	Hercynine	[M-H]
180.1027	-1.682	$C_{10}H_{14}NO_2^-$	3,4-Dimethoxyphenethylamine	[M-H]
208.1091	-0.245	$C_{10}H_{14}N_3O_2^-$	4-[Methyl(nitroso)amino]-1-(3-pyridinyl)-1-butanol	[M-H]
224.104	-0.294	$C_{10}H_{14}N_3O_3^-$	4-(methylnitrosamino)-1-(3-pyridyl-n-oxide)-1-butanol	[M-H]
183.1137	-1.103	$C_9H_{15}N_2O_2^-$	N-(3-acetamidopropyl)pyrrolidin-2-one	[M-H]

195.1137	-1.035	$C_{10}H_{15}N_2O_2^-$	2-Isopropyl-3,5-dimethoxy-6-methylpyrazine	[M-H]
223.1085	-1.420	$C_{11}H_{15}N_2O_3^-$	Butalbital	[M-H]
201.1358	0.498	$C_8H_{17}N_4O_2^-$	N,N-dimethylarginine	[M-H]
209.1293	-1.205	$C_{11}H_{17}N_2O_2^-$	Cyclo(leucylprolyl)	[M-H]
211.145	-0.956	$C_{11}H_{19}N_2O_2^-$	1,4-Bipiperidine-1-carboxylic acid	[M-H]
198.1611	-0.433	$C_{10}H_{20}N_3O^-$	Diethylcarbamazine	[M-H]
228.1716	-0.661	$C_{11}H_{22}N_3O_2^-$	N-{3-[(4-Acetamidobutyl)amino]propyl}acetamide	[M-H]
311.1685	-0.450	$C_{17}H_{27}O_3S^-$	4-Undecylbenzenesulfonic acid	[M-H]
299.2014	-0.849	$C_{20}H_{27}O_2^-$	Tretinoin	[M-H]
253.2174	0.380	$C_{16}H_{29}O_2^-$	Palmitelaidic acid	[M-H]
325.1839	-1.199	$C_{18}H_{29}O_3S^-$	4-Dodecylbenzenesulfonic acid	[M-H]
303.2327	-0.837	$C_{20}H_{31}O_2^-$	Icosa-5,8,11,14-tetraenoic acid	[M-H]
269.2488	0.728	$C_{17}H_{33}O_2^-$	Margaric acid	[M-H]
305.2483	-0.996	$C_{20}H_{33}O_2^-$	eicosa-8,11,14-trienoic acid	[M-H]
283.2642	-0.190	$C_{18}H_{35}O_2^-$	Octadecanoic acid/ Stearic acid	[M-H]
766.5398	0.745	$C_{43}H_{77}NO_8P^-$	1-stearoyl-2-arachidonoyl-sn-glycero-3-phosphoethanolamine (PE38:4)	[M-H]
744.555	0.163	$C_{41}H_{79}NO_8P^-$	1-stearoyl-2-oleoyl-sn-glycero-3-phosphoethanolamine zwitterion	[M-H]
883.5344	0.221	$C_{47}H_{80}O_{13}P^-$	1-oleoyl-2-arachidonoyl-sn-glycero-3-phospho-1D-myo-inositol	[M-H]
885.549	-0.965	$C_{47}H_{82}O_{13}P^-$	1-stearoyl-2-arachidonoyl-sn-glycero-3-phospho-1D-myo-inositol (PI 38:4)	[M-H]

Table S3. Processed data normality tests

Variable	Shapiro-Wilk Statistic	Shapiro-Wilk p-value	Kolmogorov-Smirnov Statistic	Kolmogorov-Smirnov p-value

127.0039	0.766	0	0.219	0.005
135.031	0.191	0	0.454	0
167.0208	0.67	0	0.25	0.001
85.0292	0.494	0	0.297	0
133.0145	0.879	0	0.158	0.086
149.0093	0.82	0	0.232	0.002
129.0192	0.71	0	0.24	0.001
172.9913	0.789	0	0.223	0.004
119.0171	0.874	0	0.136	0.193
115.0512	0.379	0	0.365	0
83.0499	0.393	0	0.393	0
115.0401	0.69	0	0.248	0.001
111.045	0.677	0	0.273	0
159.0301	0.859	0	0.169	0.053
203.0196	0.413	0	0.351	0
183.0119	0.379	0	0.43	0
130.0508	0.394	0	0.358	0
105.0378	0.846	0	0.18	0.033
177.0402	0.651	0	0.284	0
121.0658	0.227	0	0.479	0
241.0115	0.555	0	0.343	0
168.0431	0.786	0	0.217	0.005
179.0558	0.582	0	0.3	0
259.0224	0.538	0	0.315	0
299.2014	0.49	0	0.332	0
253.2174	0.44	0	0.371	0
303.2327	0.533	0	0.333	0
269.2488	0.558	0	0.331	0
283.2642	0.752	0	0.228	0.003
766.5398	0.36	0	0.409	0
885.549	0.576	0	0.31	0
206.9961	0.458	0	0.343	0
130.0653	0.425	0	0.396	0

173.0269	0.47	0	0.335	0
86.0966	0.563	0	0.325	0
184.0736	0.694	0	0.271	0
255.2328	0.736	0	0.232	0.002
279.2326	0.457	0	0.381	0
419.2574	0.589	0	0.299	0
437.2675	0.516	0	0.392	0
464.3142	0.407	0	0.425	0
480.3098	0.525	0	0.364	0
616.4721	0.506	0	0.405	0
905.3844	0.207	0	0.476	0
718.5382	0.415	0	0.367	0
994.4794	0.686	0	0.248	0.001
861.5491	0.385	0	0.367	0
910.5677	0.801	0	0.206	0.009
833.5926	0.867	0	0.203	0.011
813.6008	0.679	0	0.292	0
711.6513	0.537	0	0.373	0
810.6464	0.805	0	0.206	0.01
1013.4918	0.776	0	0.216	0.006
542.4906	0.387	0	0.373	0

Table S4. Results for classification Models

Model	Set	accuracy	f1 score	precision	recall	AUC
Logistic Regression	Test	0.958 ± 0.052	0.953 ± 0.060	0.967 ± 0.067	0.950 ± 0.100	0.955 ± 0.056

Logistic Regression	Train	1.000 ± 0.000	1.000 ± 0.000	1.000 ± 0.000	1.000 ± 0.000	1.000 ± 0.000
Random Forest	Test	0.920 ± 0.098	0.933 ± 0.082	0.886 ± 0.140	1.000 ± 0.000	0.920 ± 0.098
Random Forest	Train	1.000 ± 0.000	1.000 ± 0.000	1.000 ± 0.000	1.000 ± 0.000	1.000 ± 0.000
SVM	Test	0.858 ± 0.135	0.862 ± 0.122	0.867 ± 0.163	0.870 ± 0.108	0.855 ± 0.135
SVM	Train	0.854 ± 0.035	0.858 ± 0.033	0.842 ± 0.044	0.875 ± 0.027	0.854 ± 0.034
XGBoost	Test	0.853 ± 0.083	0.861 ± 0.082	0.846 ± 0.130	0.920 ± 0.160	0.860 ± 0.080
XGBoost	Train	0.881 ± 0.061	0.877 ± 0.079	0.894 ± 0.098	0.895 ± 0.163	0.879 ± 0.064

Table S5. Logistic regression average coefficients and statistical significance. Rows which are highlighted in yellow, indicates variable (m/z) that were deemed significant (p <0.05) and displayed > x2 fold change from Volcano plot. Column highlighted in grey, shows variables that displayed statistical significance (p <0.05). Negative mean coefficient indicates metabolites that are more associated with catheter surface at 1 day while positive mean coefficient indicates metabolites that are more associated with catheter surface at 28 days.

Variable	Assignment	Adduct	Mean Coefficient	Std Coefficient	P-Value
179.0558	myo-Inositol/ Fructose/ Glucose/ Galactose/ beta-D-Galactopyranose	[M-H]	-0.00046	5.76E-05	0
119.0171	4-Thiapentanoic acid	[M-H]	-0.00034	5.12E-05	0
85.0292	3-Butenoic acid/ Biacetyl/ butyrolactone/ oxolan-3-one	[M-H]	-0.0003	5.37E-05	0
241.0115	Inositol cyclic phosphate	[M-H]	-0.00025	0.000107	0.006
279.2326	FA 18:2	[M-H]	-0.00023	0.000086	0.003
133.0145	Malate/ (±)-Malic Acid	[M-H]	-0.00022	6.78E-05	0.002
833.5926	PG 39:1;O	[M-H]	-0.00021	0.000134	0.025
129.0192	Itaconate/ Mesaconate/ (Z)-glutaconic acid/ 4,5-dioxovaleric acid	[M-H]	-0.00018	6.16E-05	0.002
173.0269	p-Toluenesulfonic acid	[M+H]	-0.00017	0.00012	0.032
115.0401	3-Methyl-2-oxobutanoic acid/ Levulinic acid	[M-H]	-0.00014	0.000031	0
111.045	Sorbic acid	[M-H]	-0.00013	2.98E-05	0
885.549	PI 38:4	[M-H]	-0.00013	5.96E-05	0.008
177.0402	L-Gulono-14-lactone/ D-Glucono-14-lactone	[M-H]	-0.0001	2.38E-05	0
86.0966	Piperidine	[M+H]	-7.1E-05	3.23E-05	0.007
861.5491	PI 36:2/PI O-36:3;O	[M-H]	-6.8E-05	3.92E-05	0.017
283.2642	Octadecanoic acid/ Stearic acid	[M-H]	-6.6E-05	2.35E-05	0.003
184.0736	Choline phosphate	[M+H]	-5.1E-05	8.69E-05	0.256

130.0508	5-Aminolevulinate/ 1759032/ Ac-Ala-OH/ Aminolevulinic acid/ L-Hydroxyproline	[M-H]	-4.3E-05	8.56E-06	0
130.0653	leucoline	[M+H]	-4.2E-05	2.18E-05	0.012
172.9913	Phenol sulfate	[M-H]	-3.6E-05	3.23E-05	0.065
259.0224	D-Fructose 6-phosphate/ D-glucose 6-phosphate/ D-glucopyranose 6-phosphate	[M-H]	-3.5E-05	1.16E-05	0.002
419.2574	LPA O-18:2	[M-H]	-2.7E-05	1.93E-05	0.035
299.2014	Tretinoin	[M-H]	-2.6E-05	8.71E-06	0.002
206.9961	Pyrogallol-2-O-sulphate	[M+H]	-1.4E-05	1.14E-05	0.048
203.0196	2,6-Anhydro-6-carboxy-3-deoxyhex-2-enonic acid	[M-H]	-1.4E-05	1.9E-06	0
115.0512	1-Nitroso-3-pyrrolidinol/ Succinamide	[M-H]	-9.3E-06	3.95E-06	0.006
183.0119	4-styrenesulfonic acid	[M-H]	-8.4E-06	1.57E-05	0.299
464.3142	LPC O-15:1/LPE O-18:1	[M-H]	-6.3E-06	3.8E-06	0.02
437.2675	LPA 18:0/LPA O-18:1;O	[M-H]	-2.2E-06	3.16E-06	0.193
83.0499	Cyclopentanone	[M-H]	-1.9E-06	8.71E-06	0.646
149.0093	(.+/-.)-Tartaric acid	[M-H]	8.95E-07	4.82E-07	0.014
135.031	Hypoxanthine	[M-H]	2.06E-06	8.65E-06	0.623
718.5382	CerP 39:1;O4/LPC 31:1;O/LPE 34:1;O/LPS O-33:1/PC 31:0/PC O-31:1;O/PE 34:0/PE O-34:1;O	[M-H]	2.66E-06	3.25E-06	0.141
616.4721	CerP 34:1;O2/LPC O-26:2/LPE O-29:2	[M-H]	3.53E-06	2.07E-06	0.019
711.6513	DG O-41:0;O2	[M-H]	7.64E-06	6.58E-06	0.06
813.6008	PA 43:2;O/PG O-40:3	[M-H]	1.13E-05	8.77E-06	0.045
810.6464	HexCer 41:2;O3	[M-H]	1.13E-05	1.11E-05	0.085
121.0658	Phenylethyl alcohol	[M-H]	1.14E-05	2.22E-05	0.315
269.2488	Margaric acid	[M-H]	1.14E-05	3.14E-06	0.001
766.5398	PE 38:4	[M-H]	1.27E-05	8.06E-06	0.024
480.3098	LPC 15:0/LPC O-15:1;O/LPE 18:0/LPE O-18:1;O	[M-H]	1.87E-05	4.57E-06	0
127.0039	5-Hydroxy-2-furoic acid	[M-H]	1.96E-05	1.28E-05	0.026
910.5677	PI-Cer 39:2;O6	[M-H]	2.25E-05	7.18E-06	0.002
994.4794	MIPC 34:6;O6	[M-H]	2.96E-05	1.13E-05	0.004
253.2174	Palmitelaidic acid	[M-H]	0.000031	1.46E-05	0.008
303.2327	Icosa-5,8,11,14-tetraenoic acid	[M-H]	3.52E-05	3.31E-05	0.076
1013.492	PIP 39:5	[M+Cl]	3.63E-05	2.08E-05	0.017
542.4906	Cer 34:2;O/NAE 32:2	[M+Na]	3.66E-05	2.94E-05	0.049
105.0378	Methionol	[M-H]	3.97E-05	1.95E-05	0.01
159.0301	2-Oxoadipate	[M-H]	4.34E-05	1.57E-05	0.003
167.0208	Urate/ Uric Acid	[M-H]	0.00014	0.000099	0.034
905.3844	PIP 33:7;O	[M-H]	0.000204	0.000134	0.027
255.2328	FA 16:0	[M-H]	0.000336	7.37E-05	0
168.0431	P-DMEA	[M-H]	0.000383	7.99E-05	0

Table S6. Molecules present on the surface of the deposit (close to the interface) categorised as either interfacial or distant from interface at 1 day and 28 days

1 day	28 days
**Interfacial**	**Interfacial**
• Succinic anhydride<br>• Xylonic acid<br>• myo-Inositol/ Fructose/ Glucose/ Galactose/ Galactopyranose<br>• Itaconate/ Mesaconate/ Glutaconic acid/ 4,5-dioxovaleric acid<br>• Inositol cyclic phosphate<br>• *PIP 39:5*<br>• *FA 18:2*<br>• *PI 38:4*<br>• *FA 18:1*<br>• *FA 18:0*<br>• *LPA O-18:2*<br>• *Eicosatetraenoic acid*<br>• *FA 16:0*<br>• *Hex2Cer 34:4;O4*	• Inositol cyclic phosphate<br>• Cytosine<br>• Thymine<br>• Uracil<br>• Adenine<br>• Guanine<br>• L-Proline<br>• *FA 16:0*<br>• *FA 18:1*<br>• *FA 18:0*<br>• *Eicosatetraenoic acid*<br>• *LPA O-18:2*<br>• *Hex2Cer 34:4;O4*<br>• *PI 38:4*
**Distant from interface**	**Distant from interface**
• Cytosine<br>• Thymine<br>• Uracil<br>• Adenine<br>• Guanine<br>• L-Proline<br>• *SHexCer 41:5;O5*<br>• *PG 39:1;O*	• *SHexCer 41:5;O5*<br>• *PG 39:1;O*<br>• *PIP 39:5*<br>• *FA 18:2*

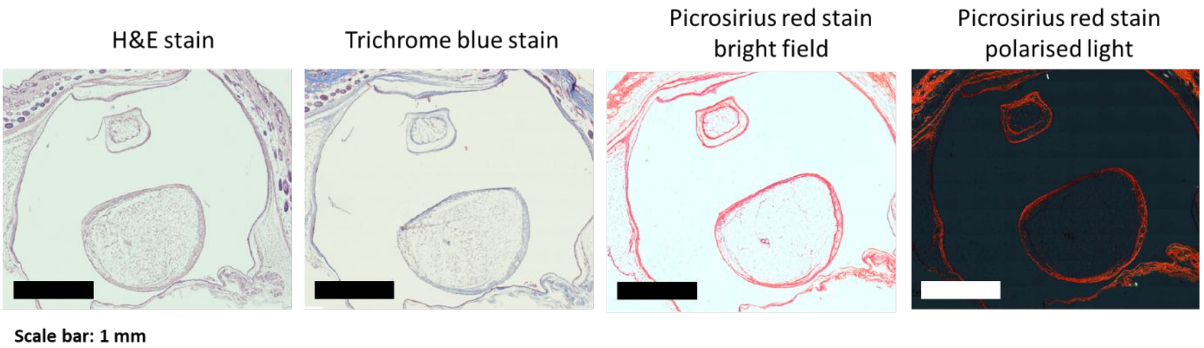

Figure S1. The histological stain of the surrounding tissue. The tissue were stained with Haematoxylin and eosin (H&E), Masson's trichrome stain and picrosirius red stain

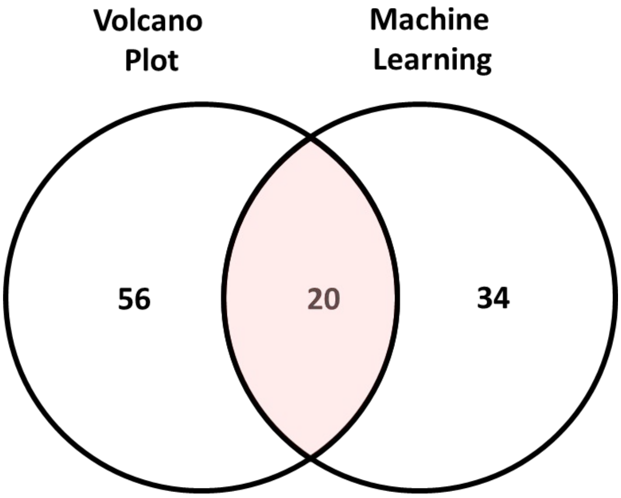

Figure S2. The Venn diagram shows the number of assignments overlap between the logistic regression analysis with the univariate statistics Volcano plot approach.

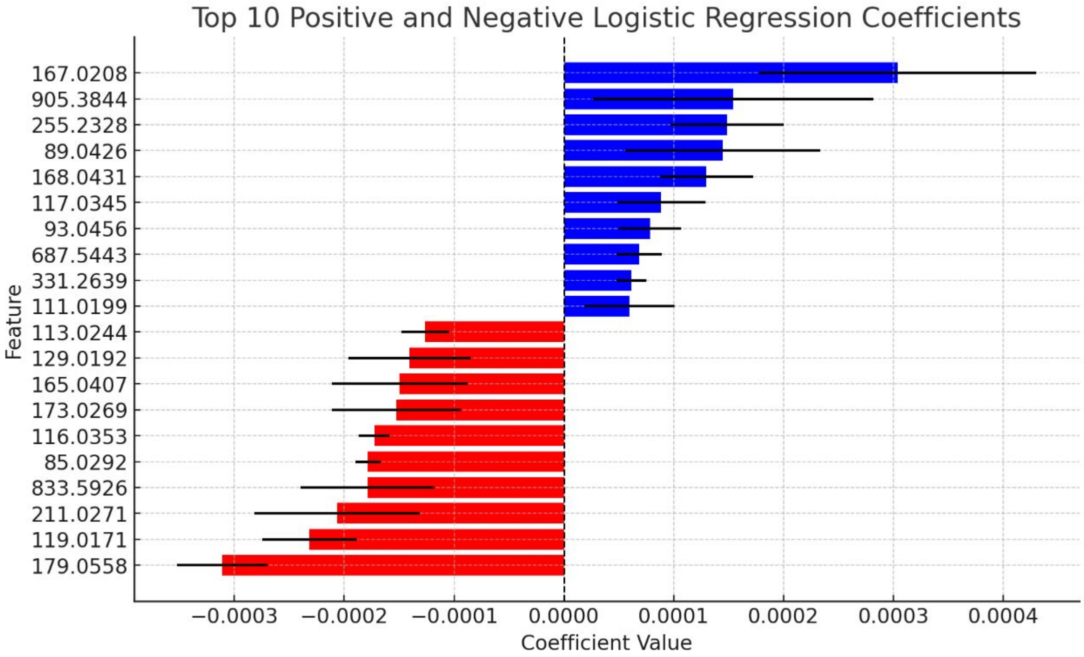

Figure S3. Top ten positive and top ten negative logistic regression coefficients

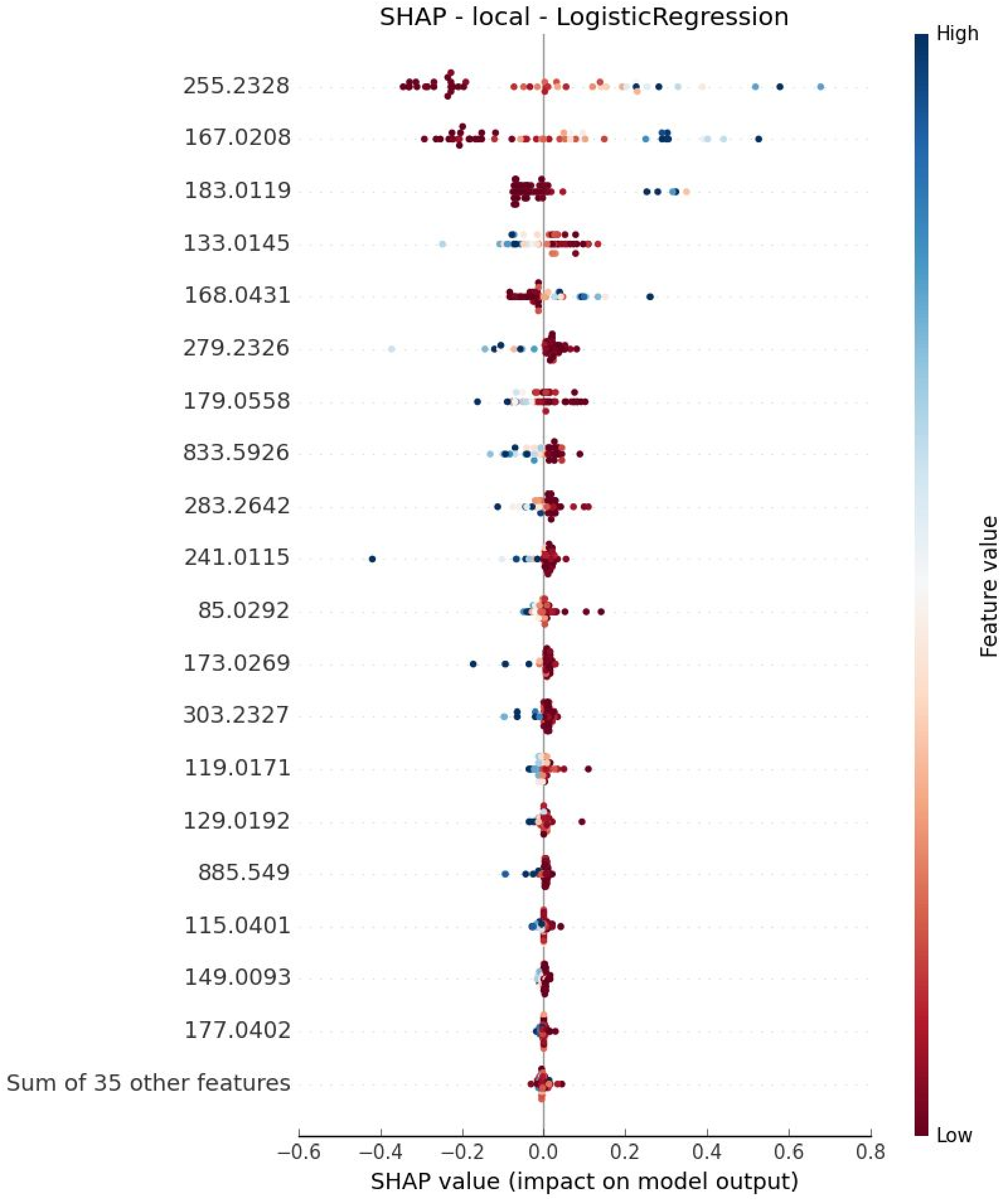

Figure S4. Shapley (SHAP) importance values for Logistic Regression

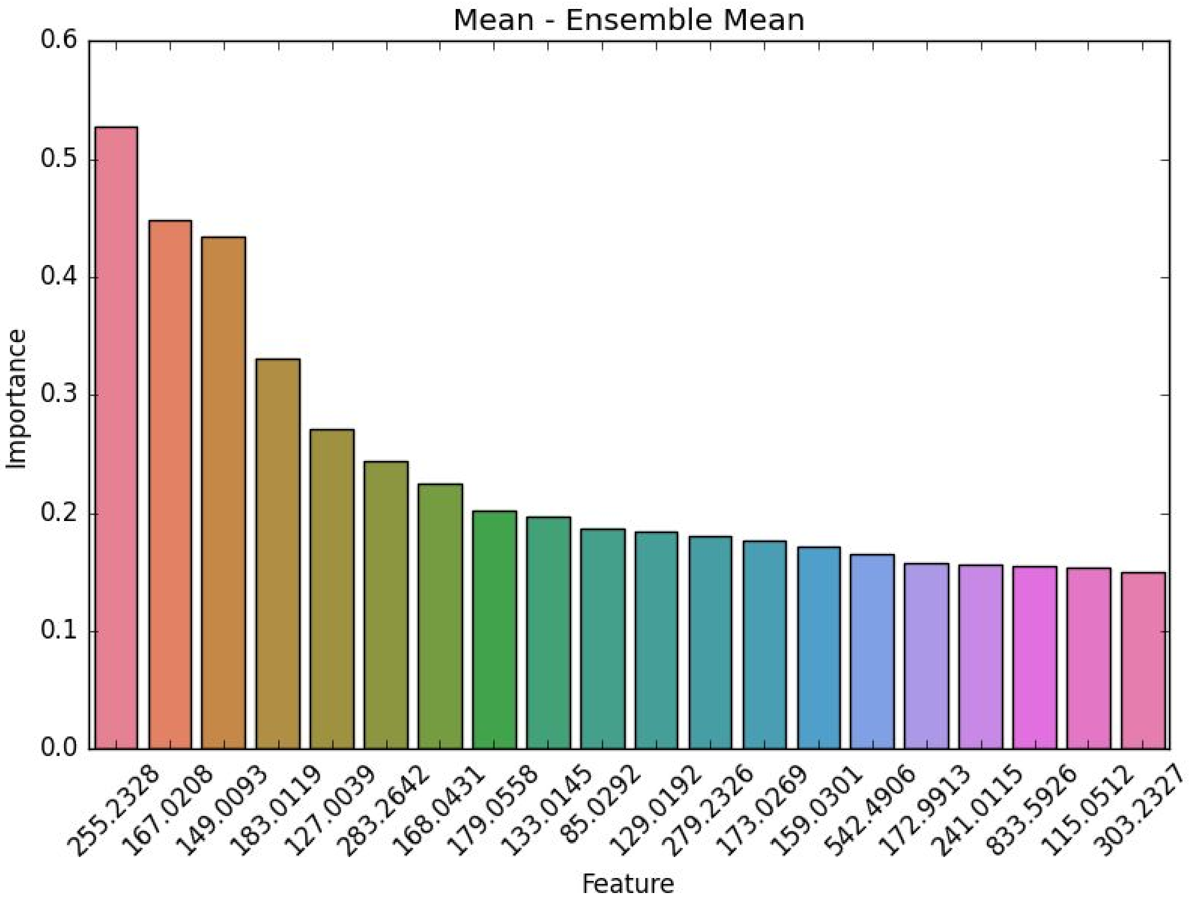

Figure S5. Average feature importance across modelling approaches

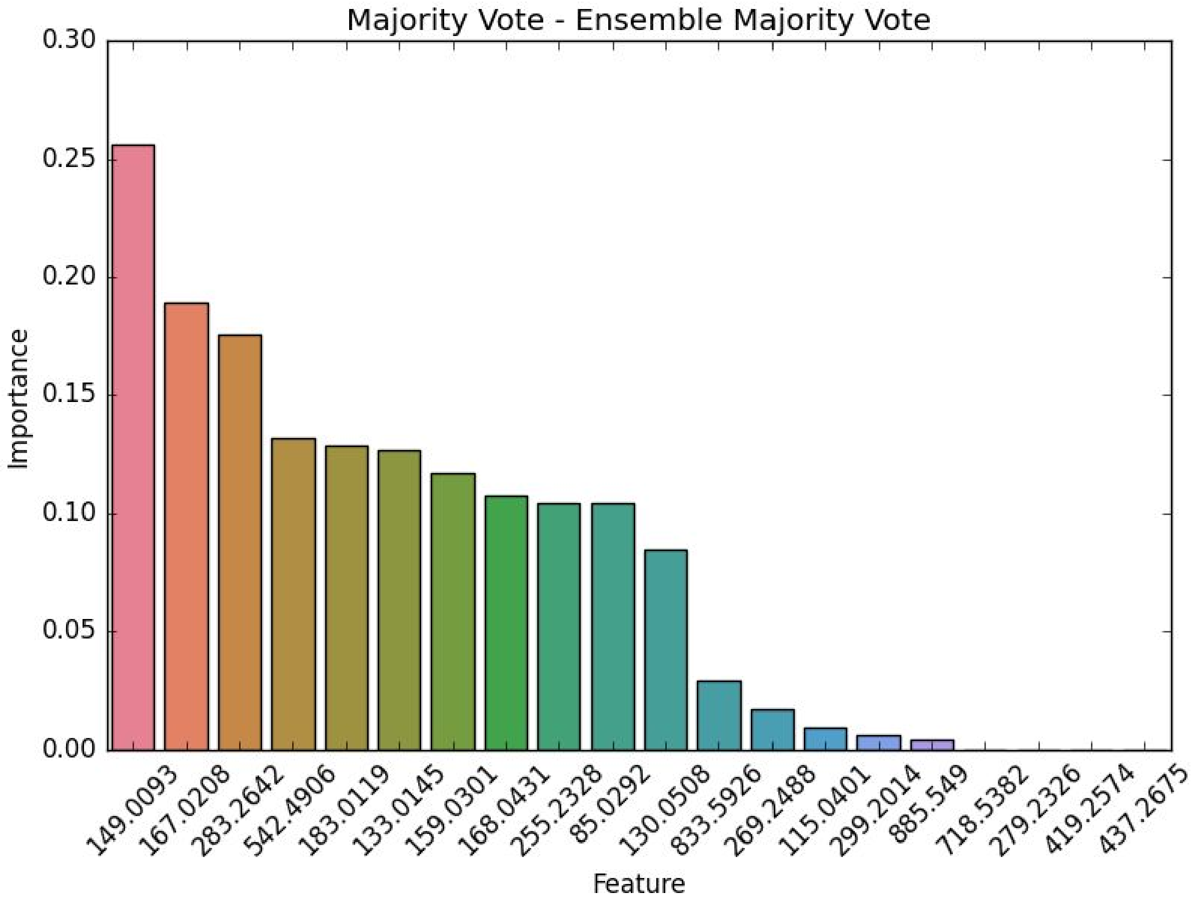

Figure S6. Majority voting results determining feature importance across modelling approaches

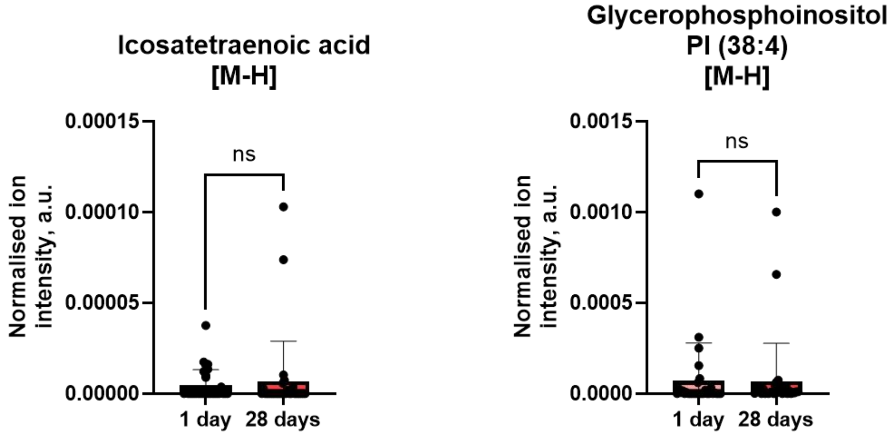

Figure S7. Bar charts of normalised ion intensity of icosatetraenoic acid and glycerophosphoinositol PI (38:4). Data are plotted as normalised ion intensities to total ion count. Data are displayed as n=30, mean ± SD.

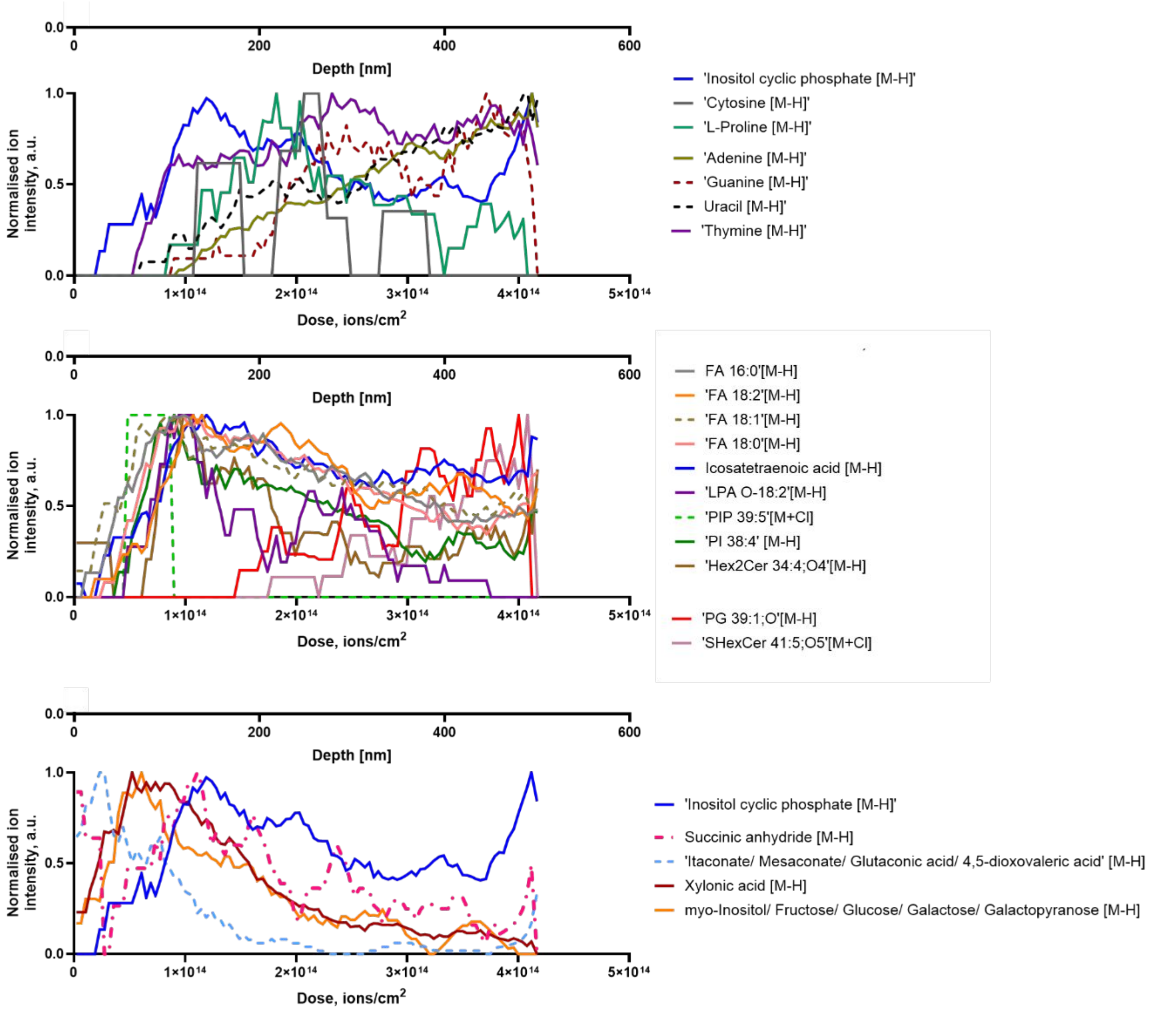

Figure S8. Depth profile at silicone catheter surfaces versus primary ions dose and estimated depth displaying lipids, amino acid, nucleic acid bases and sugar-based metabolites molecular ions profile at 1 day.

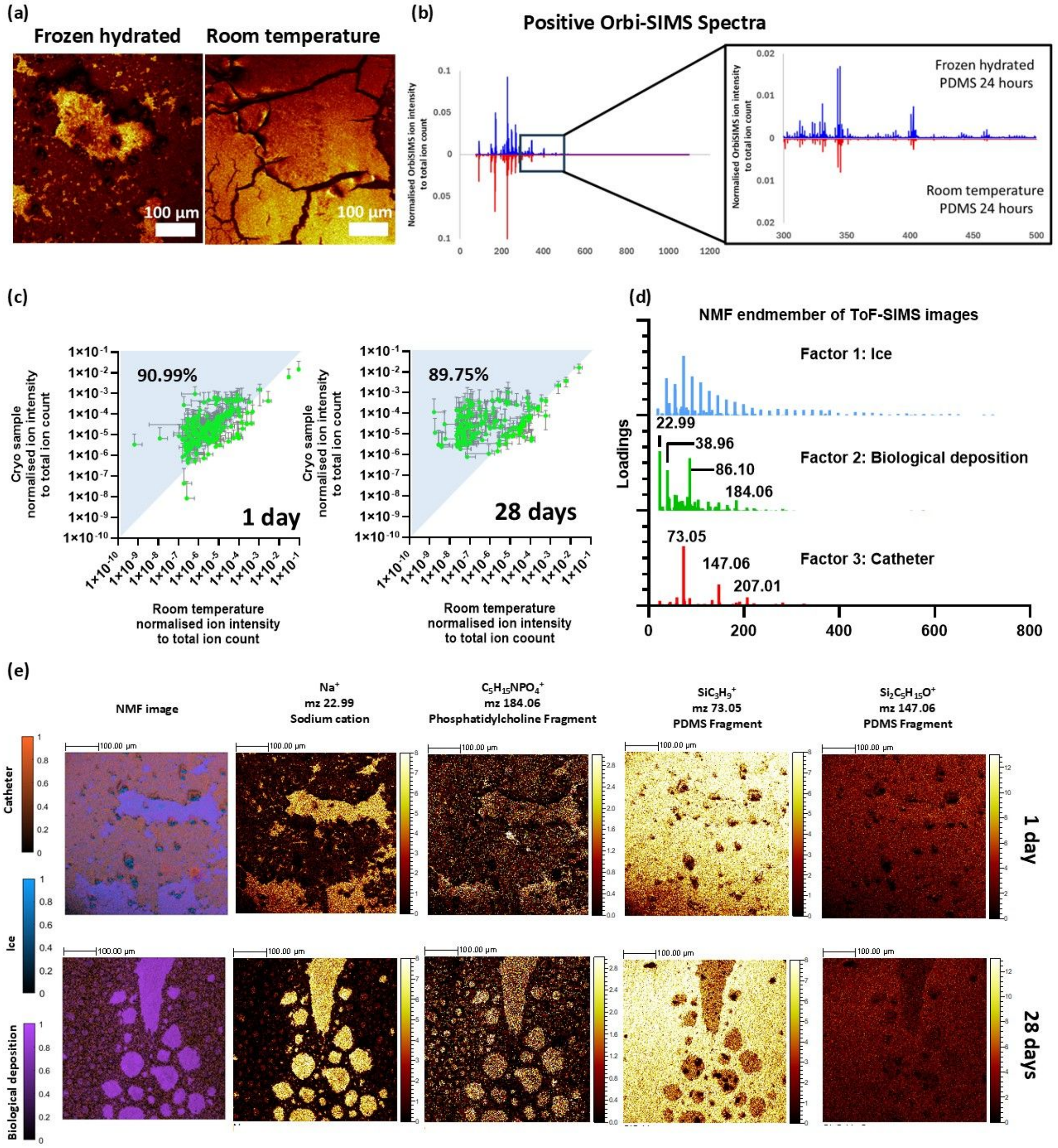

Figure S9 (a) LMIG/ToF-SIMS ion images of the catheter surfaces when sample were analysed under frozen hydrated and room temperature conditions. (b) Comparison of GCIB/OrbiSIMS spectra of catheter surfaces analysed under frozen-hydrated (blue) and room temperature (red) conditions. (c) Scatter plot comparing the normalised ion intensities of biomolecules detected when the surface of the catheters was analysed under room temperature and frozen hydrated. (d) Non-negative matrix factor (NMF) loadings showing the separation of LMIG/ToF-SIMS data into three distinct components: ice, biological deposition, and catheter with (e) representative NMF images for 1- and 28-days implantation following 500 NMF iterations. The NMF analysis was performed using loadings and images from raw LMIG/ToF datasets. Colour scale represents the intensity of NMF endmembers post calculation along with the ion images for C5H15NPO4+ which represent phosphatidylcholine fragments and SiC3H9+ and Si2C5H15O+ which is used as a marker for PDMS. Scale bar: 100 µm.

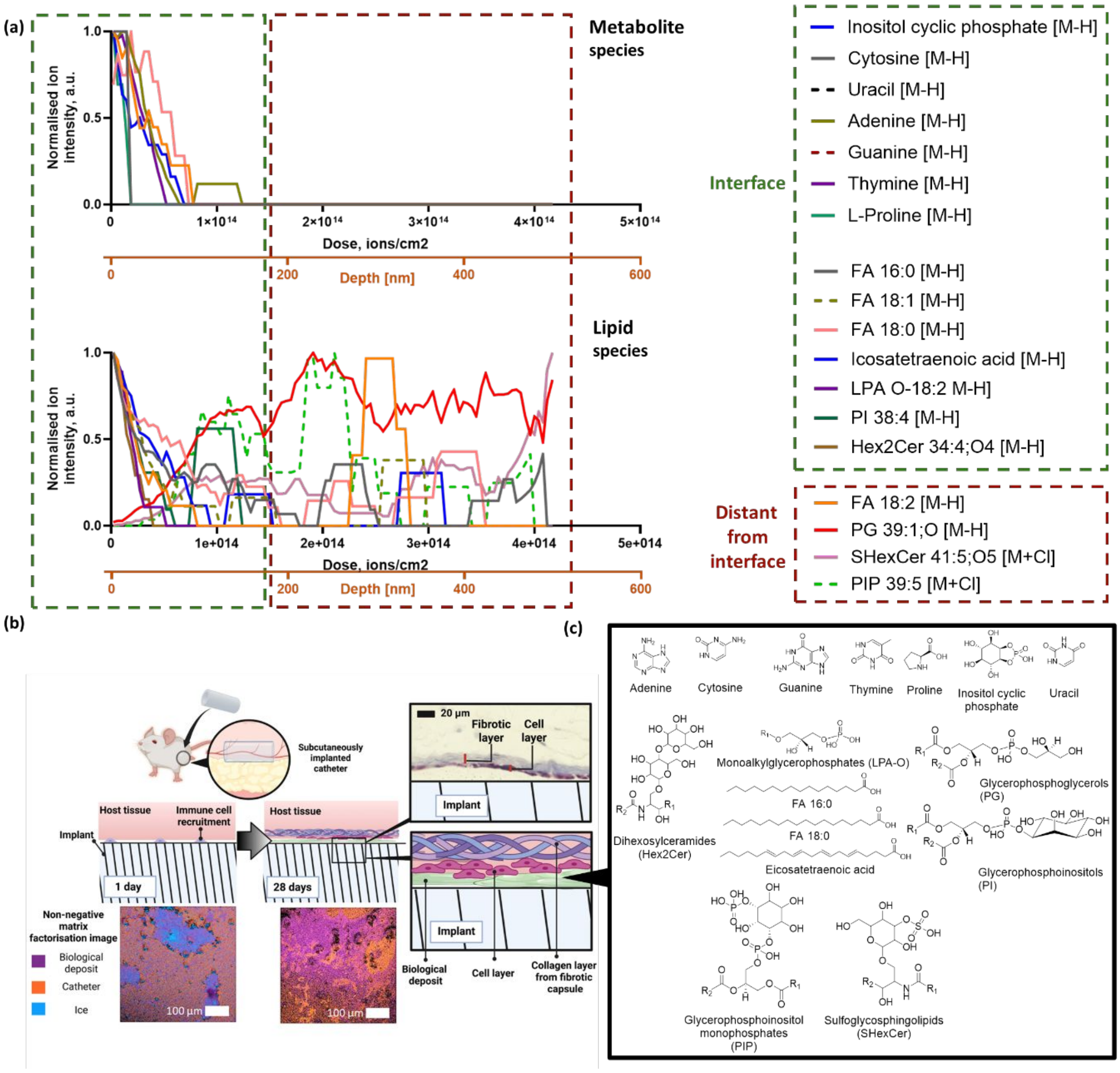

Figure S10 (a) Depth profile at silicone catheter surfaces versus primary ions dose (ions/cm^2) and estimated depth displaying lipids, amino acid and nucleic acid bases molecular ions at 28 days. Molecules present on the surface of the deposit (close to the interface) is categorised as interfacial while those present deep in the deposit is categorised as distant from interface. The secondary x axis presents depth estimated from comparison with organic standards **(b)** Schematic illustrating and summarising the presence of biological deposit formed on the surface of medical device following implantation **(c)** Chemical structure of species detected by cryo-OrbiSIMS.

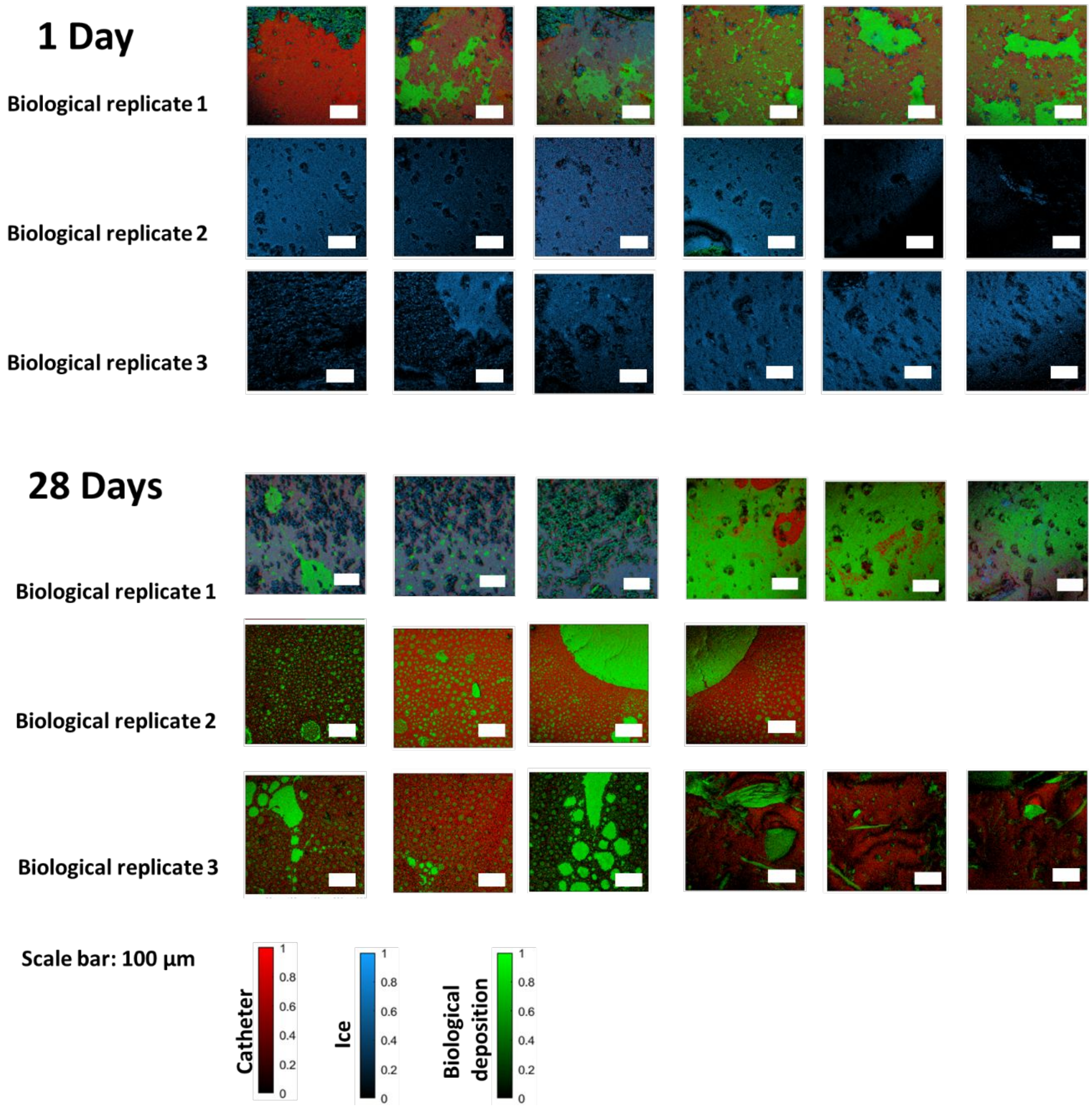

Figure S11. NMF Images of catheter explant collected Balb/c mice at 1 day and 28 days. Colour scale represents the intensity of NMF endmembers post calculation The absence or minimal signal from catheter or biological deposits for these NMF images for biological replicate 2 and 3 was attributed to the formation of ice on the surface of the catheter during sample analysis. The formation of ice on the surface prevents from any further ToF-SIMS data analysis, however this does not impact the Orbi-SIMS data acquisition

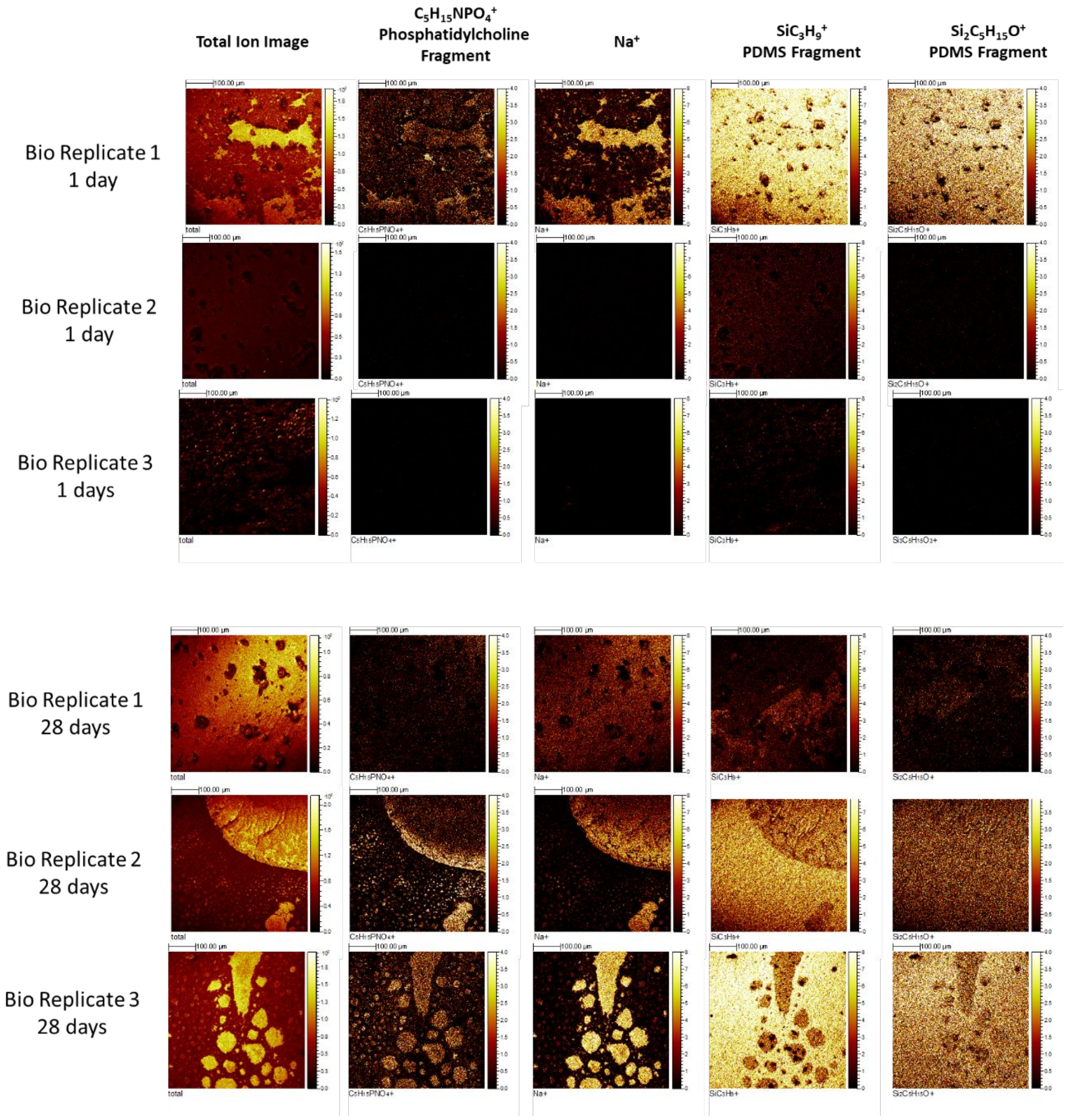

Figure S12. ToF-SIMS chemical ion images of catheter explant collected Balb/c mice at 1 day and 28 days. The ion images selected are $C_5H_{15}NPO_4^+$ Phosphatidylcholine fragments and Na^+ which are used as markers for biological deposits for ToF images and $Si_2C_5H_{15}O^+$ which is used as a marker for PDMS oligomer fragment. The absence or minimal signal for these ion images for biological replicate 2 and 3 was attributed to the formation of ice on the surface of the catheter during sample analysis. The formation of ice on the surface prevent from any further ToF-SIMS data analysis, however this does not impact the Orbi-SIMS data acquisition.
